# Comprehensive molecular profiling of urologic tumors presented in a molecular tumor board: insights from a real-world precision oncology cohort

**DOI:** 10.3389/fonc.2025.1688779

**Published:** 2025-11-27

**Authors:** Maximilian Glienke, Ruth Himmelsbach, Max Zirngibl, Patrick Metzger, Julia Kühn, Cornelius Miething, August Sigle, Christian Gratzke, Cathleen Nientiedt, Michael Rassner, Heiko Becker, Justus Duyster, Silke Lassmann, Martin Werner, Melanie Börries, Markus Grabbert

**Affiliations:** 1 Department of Urology, Medical Center, Faculty of Medicine, University of Freiburg, Freiburg, Germany; 2 Comprehensive Cancer Center Freiburg, Medical Center, Faculty of Medicine, University of Freiburg, Freiburg, Germany; 3 Institute of Medical Bioinformatics and Systems Medicine, Medical Center, Faculty of Medicine, University of Freiburg, Freiburg, Germany; 4 Molecular Tumorboard Network (MTB) Freiburg, Freiburg, Germany; 5 German Cancer Consortium (DKTK), Partner Site Freiburg of the German Cancer Research Center (DKFZ), Heidelberg, Germany; 6 Department of Medicine I, Medical Center, Faculty of Medicine, University of Freiburg, Freiburg, Germany; 7 Institute for Surgical Pathology, Medical Center, Faculty of Medicine, University of Freiburg, Freiburg, Germany

**Keywords:** urologic oncology, molecular tumor board, precision medicine, prostate cancer, bladder cancer, renal cell carcinoma

## Abstract

**Objectives:**

Molecular tumor boards (MTBs) have become an integral component of precision oncology, yet data on their real-world impact in urologic cancers are limited. This study aimed to characterize the molecular landscape of urologic malignancies presented to the MTB at the University Medical Center Freiburg and to evaluate the frequency and clinical relevance of genomic alterations across tumor entities.

**Methods:**

We retrospectively analyzed 118 patients with histologically confirmed urologic tumors presented at the Freiburg MTB between Januar 2019 and December 2024. Comprehensive molecular profiling was performed using next-generation sequencing (TruSight Oncology 500 or whole exome sequencing). Data were analyzed for mutation frequency, tumor mutational burden (TMB), and co-occurrence patterns, and integrated with clinical data to guide therapy recommendations.

**Results:**

Somatic mutations were identified in 90.6% of cases. Frequent alterations included TP53, BRCA2, KMT2D, and ATM, with DNA damage response and chromatin remodeling pathways commonly affected. Prostate cancers showed high rates of BRCA2 and APC co-mutations, indicating potential benefit from combined PARP and Wnt-targeted therapies. In bladder and upper tract urothelial carcinomas (UTUC), KMT2C co-occurred with genes such as SPTA1 and LRP1B, suggesting a hypermutated, immunoresponsive phenotype. Renal tumors frequently harbored alterations in VHL, PBRM1, and SETD2. Rare entities such as penile and testicular tumors displayed distinct mutation patterns, including BRCA1/2 and MMR gene alterations.

**Conclusions:**

Comprehensive molecular profiling in a MTB setting reveals distinct and therapeutically relevant mutational patterns across urologic cancers. These data support the integration of MTBs into clinical workflows and highlight the potential of co-mutational signatures to guide personalized treatment strategies.

## Introduction

Urologic malignancies such as prostate, bladder, and renal cancer are among the most prevalent and clinically challenging cancers worldwide. In Germany, prostate cancer alone accounts for over 70,000 new cases annually and is a leading cause of cancer-related mortality in men (1). While localized disease is often manageable, advanced stages—such as castration-resistant prostate cancer, muscle-invasive bladder cancer (MIBC), and advanced renal cell carcinoma (RCC)—pose significant therapeutic challenges despite recent advances in immunotherapy and targeted agents (2, 3).

Molecular oncology has revealed considerable genetic heterogeneity across urologic cancers, including recurrent alterations in BRCA2, ATM, and VHL. These findings have enabled a shift toward precision medicine, allowing stratification of patients based on actionable molecular features (4). High-throughput platforms like next-generation sequencing (NGS) now support comprehensive tumor profiling, uncovering mutations, copy number variations, tumor mutational burden (TMB), and DNA repair deficiencies that inform prognosis and guide therapy (5).

To apply these insights clinically, molecular tumor boards (MTBs) have emerged as interdisciplinary platforms that integrate molecular data with clinical context to provide individualized treatment recommendations. MTBs are especially relevant for patients with advanced, therapy-refractory, or rare tumors (6).

Despite their growing use, data on the real-world impact of MTBs in urologic oncology are limited. This study addresses this gap by presenting the molecular landscape of urologic cancer patients evaluated at the MTB of the University Medical Center Freiburg. We analyzed both common (prostate, bladder, renal) and rare (penile, urethral, testicular, upper tract urothelial carcinomas (UTUC)) urologic cancers, with a focus on DNA repair, epigenetic, and signaling alterations of therapeutic relevance.

## Methods

### Molecular tumor board process

The Freiburg MTB convenes every two weeks and includes representatives from medical oncology, urology, pathology, human genetics, and bioinformatics. In addition, clinicians involved in clinical care of patient with diverse tumor entities present individual cases. During each session, molecular and clinical data are jointly reviewed to formulate personalized treatment recommendations. These include targeted therapies, immunotherapies, enrollment in clinical trials, or guideline-adapted treatments based on specific molecular findings.

### Patient cohort and clinical data collection

This retrospective analysis included patients with histologically confirmed genitourinary malignancies who were presented at MTB of the University Medical Center Freiburg between January 2019 and December 2024. Patients were identified via electronic health records using ICD-10 codes C60–C68, which encompass malignant neoplasms of the urinary tract and male genital organs. Inclusion criteria were: (i) a primary diagnosis of a genitourinary tumor, (ii) comprehensive molecular profiling via NGS, and (iii) formal case presentation with documented treatment recommendation at the MTB. Patients who were referred to the MTB for non-urologic tumors but had incidental urologic neoplasms were excluded from the analysis, even if molecular data were available. Clinical data—including histological subtype —were extracted from institutional records, pseudonymized, and processed in compliance with the General Data Protection Regulation (GDPR). The study protocol was approved by the Scientific Board of the Freiburg Molecular Tumor Board and patients gave informed consent to make their data available within the framework of a prospective cohort study.

### Molecular analysis

Tumor samples were analyzed using the TruSight Oncology 500 (TSO 500) panel (Illumina, Town, Country) or whole exome sequencing (WES). Immunohistochemistry (IHC) was performed where appropriate (e.g., Nectin-4, TROP2, FRα). DNA and RNA were extracted from formalin-fixed paraffin-embedded (FFPE) tumor tissue. Although the sequencing platforms used are also compatible with circulating tumor DNA (ctDNA), all samples in this urologic cohort were derived exclusively from FFPE tissue biopsies, and no liquid biopsies (ctDNA) were included in this analysis. Tissue biopsies were quality-checked (Qubit, TapeStation), and prepared following Illumina’s TSO 500 protocol (7). Libraries were pooled and sequenced on a NextSeq 550Dx. The panel covers 523 cancer-associated genes and detects Single Nucleotide Variants (SNVs), Insertion-Deletion (InDels), Copy Number Variations (CNVs), Microsatellite Instability (MSI), TMB and RNA fusions.

### Bioinformatics and variant annotation

NGS data were processed with the DRAGEN TSO 500 Local App (v2.5.2.1), aligned to hg19. Quality control included FastQC and Trimmomatic. BWA-MEM was used for alignment, with variant calling via Mutect2 (somatic) and HaplotypeCaller (germline). Annotation was performed using Ensembl-VEP and SnpEff. CNVs were adjusted for tumor cell content (Control-FREEC, Sequenza), and MSI status assessed with MSIsensor-pro. Homologous Recombination Deficiency scores were computed using scarHRD. TMB was harmonized to FoundationOne CDx/WES standards. TMB was calculated as the total number of somatic, non-synonymous mutations per sample. Due to differences in sequencing platforms and target sizes (panel-based vs. WES), TMB was not harmonized to mutations per megabase (mut/Mb) to avoid artificial precision. All reported values thus reflect absolute mutation counts per tumor. Only variants with ≥50x coverage and low population frequency (<1%) were considered. RNA fusions were validated using Arriba; known variants like AR-V7, EGFRvIII, and MET exon 14 skipping were reported independently.

Variant calling was performed using the DRAGEN TSO500 Local App (v2.5.2.1) or the GATK-based pipeline (Mutect2 for somatic and HaplotypeCaller for germline calling). Only variants with a PASS filter status were retained, ensuring that all base quality, mapping quality, strand bias, and depth-of-coverage metrics met the quality thresholds defined by the respective pipeline.

Variants were reported if they had a variant allele frequency (VAF) ≥ 5% and were supported by at least 4 variant reads.

For panel-based sequencing (TSO500), where matched normal controls were not available, variants with a population frequency > 0.1% in gnomAD were excluded to minimize the inclusion of common germline variants. This population frequency filtering serves as a proxy for germline filtering in the absence of normal control samples.

In WES datasets with available matched germline DNA, germline variants were removed bioinformatically via joint calling and subtraction. Only somatic variants were retained for downstream interpretation.

Tumor cellularity thresholds were not strictly defined. While a minimum tumor content of ~30% is generally targeted for sequencing, exceptions were made on a case-by-case basis (e.g., for rare histologies or low-input biopsies), and no uniform cutoff was enforced.

### Variant classification and prioritization

All protein-altering somatic variants that passed quality filters were included in the analysis, regardless of their clinical classification. This encompassed both clearly pathogenic or likely pathogenic variants as well as variants of uncertain significance (VUS). We excluded synonymous (silent) mutations and retained only coding variants that were predicted to alter the protein sequence.

Variants were annotated using ClinVar to assess known pathogenicity classifications and supplemented with the REVEL score, which provides a computational prediction of deleteriousness for missense variants. Additionally, variants were cross-referenced with the OncoKB Cancer Gene List to identify genes with known or putative relevance in oncogenesis.

Population frequency filtering using gnomAD ensured that common germline single nucleotide polymorphisms (SNPs) were excluded (threshold: <0.1%). No additional germline removal was performed for panel-based assays without matched normal controls. In whole-exome sequencing cases with matched normal DNA, germline variants were filtered out bioinformatically.

Final prioritization and interpretation of the variants were conducted in the context of the multidisciplinary MTB, incorporating molecular, clinical, and therapeutic relevance to guide downstream decision-making.

### Statistical analysis

Statistical analysis was conducted using R (version 4.2.0). Molecular data were imported into R and analyzed descriptively to assess the frequency and distribution of genetic alterations across tumor entities. The results were visualized using Oncoprints and interaction heatmaps.

**Figure 1:**
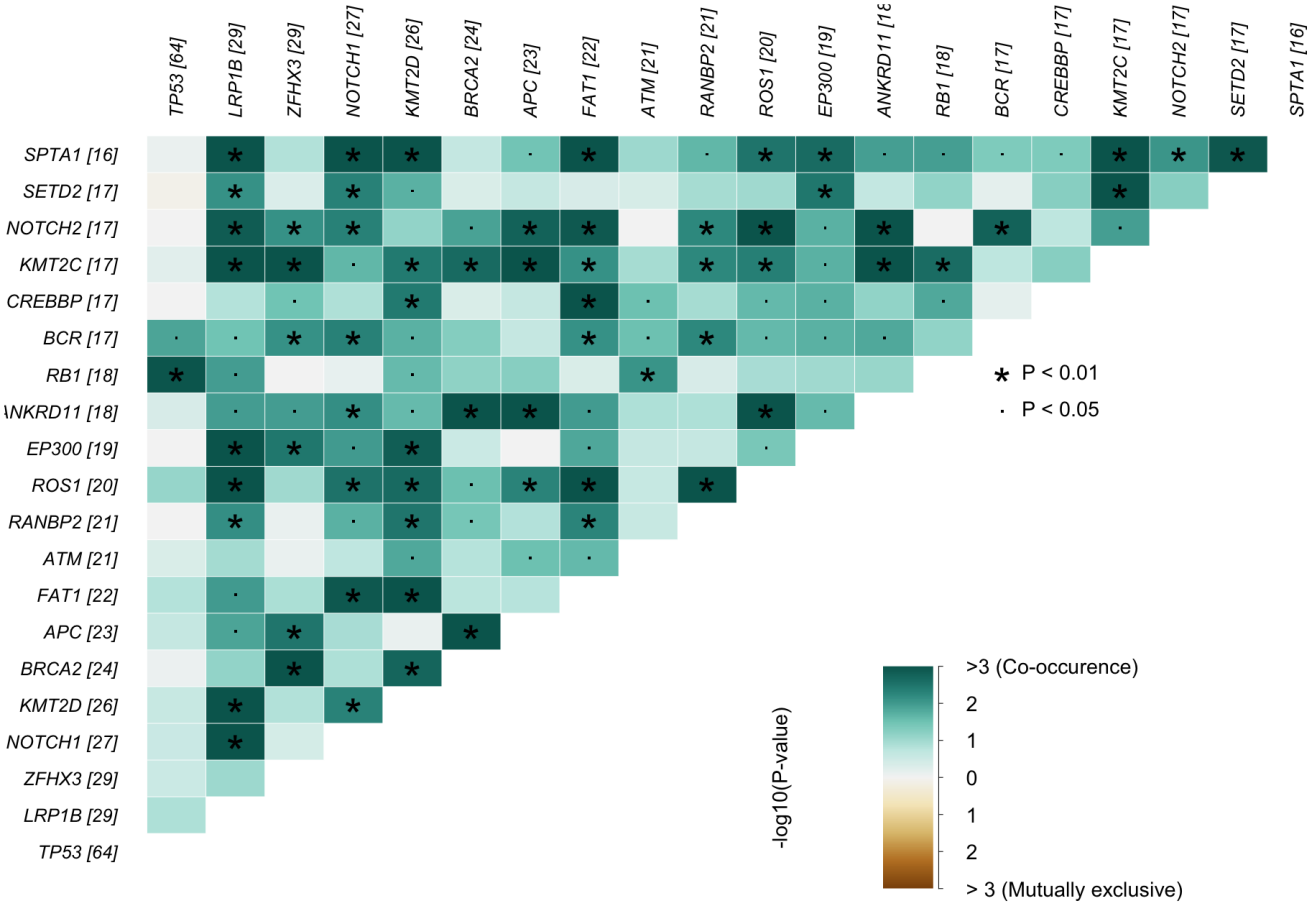
Gene–gene interaction heatmap. Lower−triangular matrix showing pairwise enrichment (co−occurrence) and depletion (mutual exclusivity) of mutations among the most frequently altered genes in the cohort (numbers in brackets indicate mutated cases per gene). Color encodes –log_10_(P) values from Fisher’s exact tests green = co−occurrence; brown = mutualexclusivity; values > 3 correspond to P < 10^−3^. All P-values were adjusted for multiple hypothesis testing using the Benjamini–Hochberg false discovery rate (FDR). Asterisks mark adjusted P < 0.01 and dots adjusted P < 0.05. Genes are ordered by decreasing mutation frequency, complementing the final Results subsection.

For co-occurrence and mutual exclusivity analysis (**Figure 1**), pairwise Fisher’s exact tests were performed using the somaticInteractions() function from the maftools package (v2.6.05). Resulting p-values were corrected for multiple testing using the Benjamini-Hochberg method (FDR), and adjusted p-values < 0.05 were considered significant.

## Results

A total of 118 patients with histologically confirmed urologic malignancies underwent comprehensive molecular profiling (**Table 1**). Across all tumor types, somatic alterations were identified in 90.6% of cases. Frequently affected genes included tumor suppressors (e.g., TP53, BRCA2, PTEN), chromatin remodelers (KMT2C/D, ARID1A/B), and DNA damage response (DDR) genes such as ATM and CHEK2. Missense mutations were the predominant variant class (>75%), with C>T transitions representing the most common SNV. TMB and mutational spectra varied considerably both across and within tumor types.

**Table 1:** Cohort characteristics of patients presented at the Molecular Tumor Board of the University Medical Center Freiburg between January 2019 and December 2024. Shown are the individual tumor subtypes, the number of cases stratified by gender, and the age distribution at presentation (mean with minimum–maximum range, in years).

Tumor	Gender		Age
	M	F	
Prostate Cancer	50		65 (22-89)
- Adenocarcinoma	43		
- Neuroendocrine Carcinoma	5		
- Rhabdomyosarcoma	1		
- Undifferentiated Carcinoma	1		
Bladder Cancer	19	11	58 (39-83)
- Urothelial Carcinoma	10	4	
- Adenocarcinoma	2	1	
- Neuroendocrine Carcinoma	4	1	
- Sarcomatoid Carcinoma		1	
- Sqamous Cell Carcinoma	2	1	
- Giant Cell Carcinoma		1	
- Urachal Adenocarcinoma		1	
- Urothelial Carcinoma of the Urethra			
- Sqamous Cell Carcinoma of the Urethra	1		
Renal Cancer	12	5	57 (19-77)
- Clear Cell Renal Cell Carcinoma	8	4	
- Papillary Renal Cell Carcinoma	2		
- Chromophobe Renal Cell Carcinoma	1		
- Collecting Duct Carcinoma		1	
- Nephroblastoma	1		
Upper Urinary Tract Urothelial Carcinoma	7	5	63 (41-78)
Testicular Cancer	7		40 (25-49)
- Seminoma	1		
- Yolk Sac Tumor	2		
- Teratoma	1		
- Chorioncarcinoma	2		
- Spermatic Cord Desmoplastic Small Round	1		
Cell Tumor			
Squamous Cell Carcinoma of the Penis	2		65 (59-71)

### Prostate cancer

**Figure 2:**
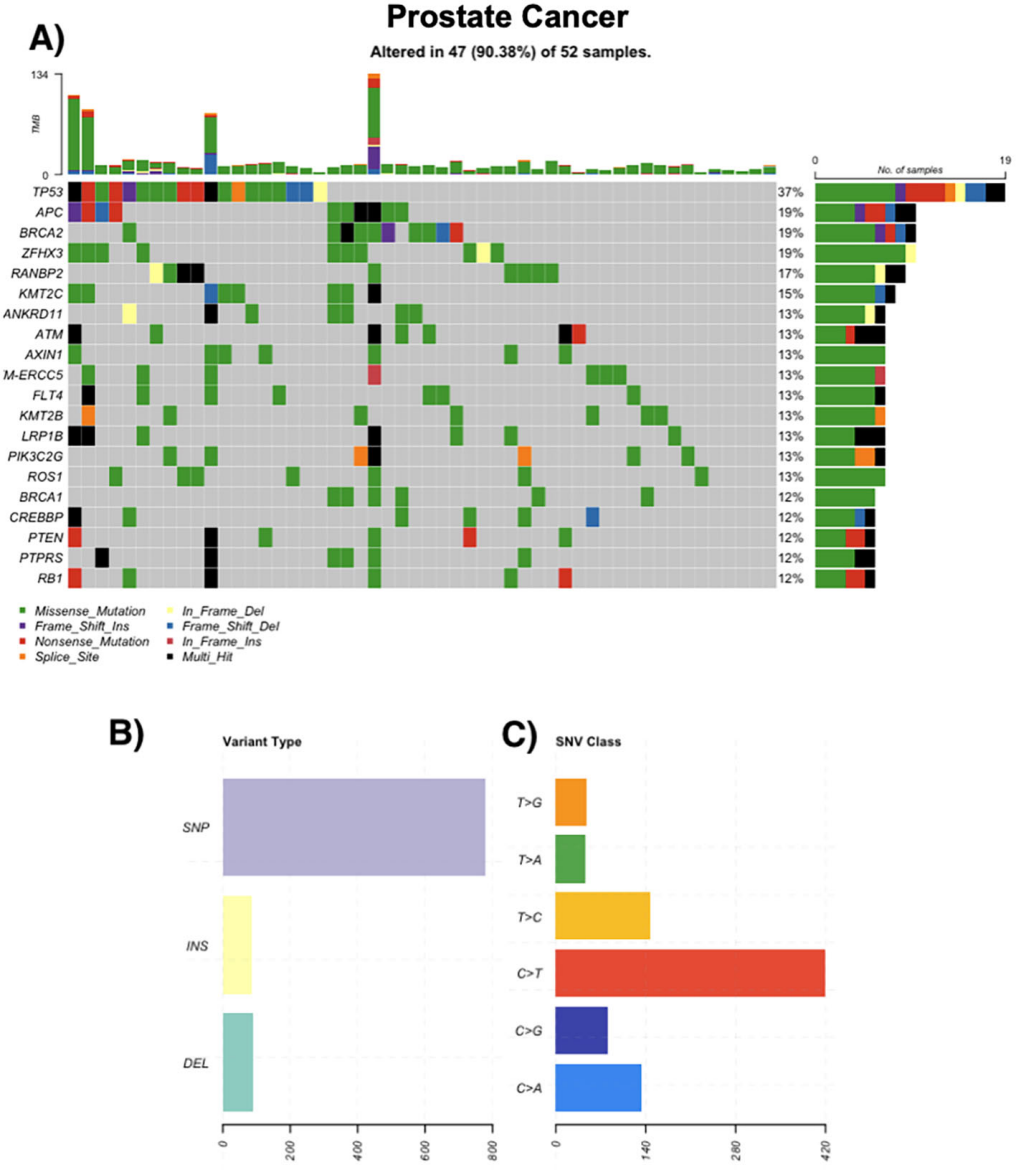
Genomic landscape of prostate cancer. **(A)** Oncoprint of the most frequently altered genes across prostate tumors (n = 52); 47/52 (90.4%) samples harbor ≥1 alteration. Tiles indicate mutation class (missense, nonsense, frameshift insertion/deletion, splice-site, in-frame insertion/deletion; legend), black denotes multi-hit events, and gray indicates no alteration. The stacked bars above columns show tumor mutational burden (TMB) per sample; right-hand bars summarize the number and percentage of altered cases per gene. **(B)** Distribution of variant types in the cohort (SNPs, insertions, deletions). **(C)** Single-nucleotide variant (SNV) spectrum by substitution class (C>A, C>G, C>T, T>A, T>C, T>G), with C>T transitions being most common.

Among 50 prostate cancer cases, 94% (n=47) had at least one somatic variant. Acinar adenocarcinoma was the most frequent subtype (n=43). Recurrently mutated genes included TP53 (37%), APC (19%), BRCA2 (19%), and ZFHX3 (19%). DDR genes such as ATM and BRCA2 were commonly altered. Variants were primarily missense SNVs, and TMB values reached up to 134 mutations/sample in individual cases. Multi-hit events were noted in TP53, PTEN, and RANBP2 (**Figure 2**).

### Renal cancer

**Figure 3:**
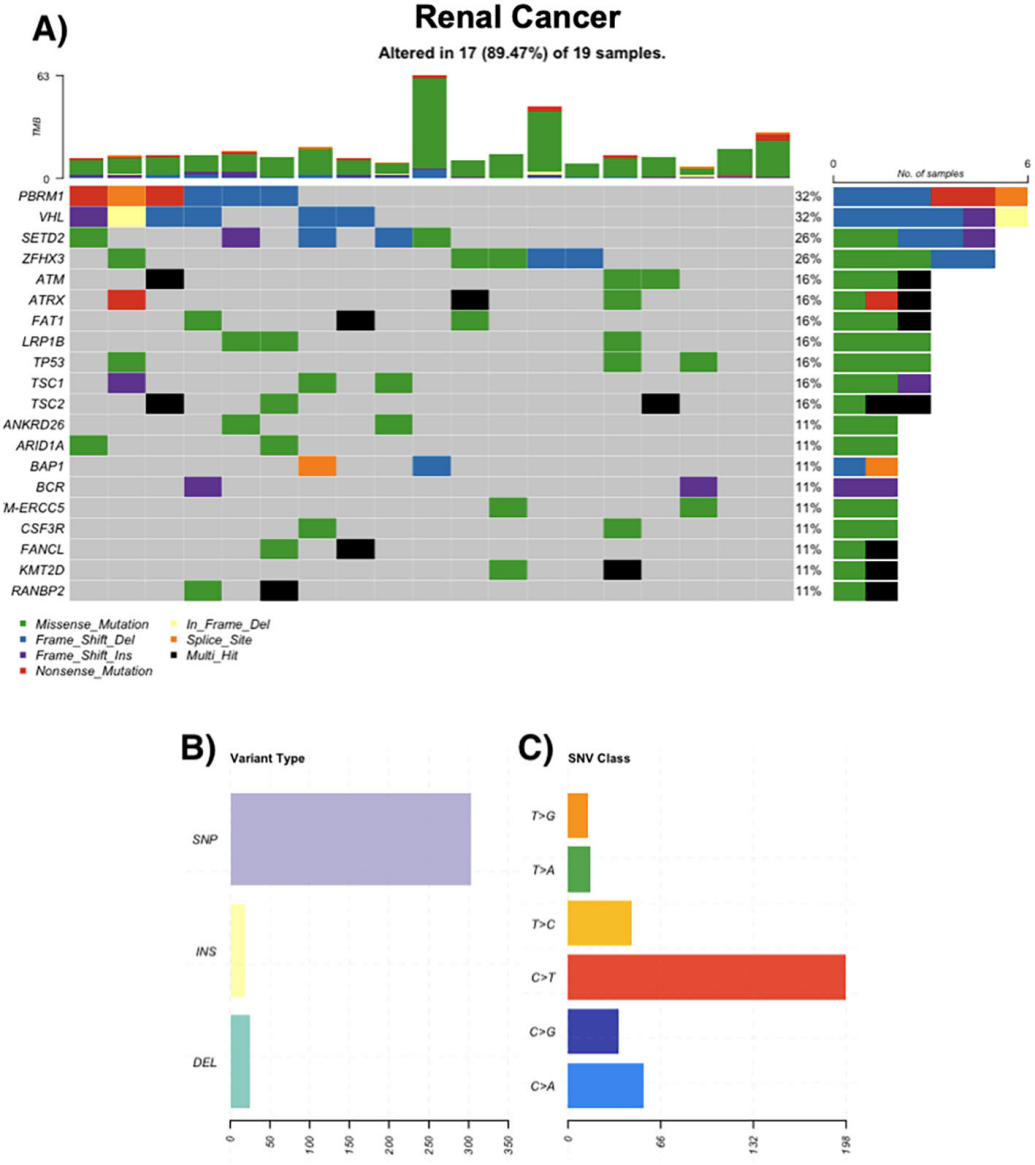
Genomic landscape of renal cancer. **(A)** Oncoprint of recurrently mutated genes in renal tumors (n = 19); 17/19 (89.47%) samples harbor ≥1 alteration. Most frequent genes: PBRM1 32%, VHL 32%, SETD2 26%, ZFHX3 26%; ATM, ATRX, FAT1, LRP1B, TP53, TSC1, TSC2 each 16%; ANKRD26, ARID1A, BAP1, BCR, ERCC5, CSF3R, FANCL, KMT2D, RANBP2 each 11%. Tiles are colored by mutation class (legend); black indicates multi-hit events and gray indicates no alteration. The stacked bars above columns show tumor mutational burden (TMB) per sample; right-hand bars summarize the number and percentage of altered cases per gene. **(B)** Distribution of variant types in the cohort, grouped as single-nucleotide polymorphisms (SNPs), insertions (INS), and deletions (DEL). **(C)** Single-nucleotide variant (SNV) spectrum by substitution class (C>A, C>G, C>T, T>A, T>C, T>G); C>T transitions are most frequent.

All 17 renal cancer cases displayed somatic alterations. Clear cell RCC predominated, followed by papillary and rare subtypes such as chromophobe RCC and collecting duct carcinoma. The most frequently altered genes were VHL (32%), PBRM1 (32%), and SETD2 (26%). Additional mutations in TSC1, TSC2, ATM, and FAT1 were observed in 16% of cases. Some tumors exhibited elevated TMBs and frameshift mutations (**Figure 3**).

### Bladder cancer

**Figure 4:**
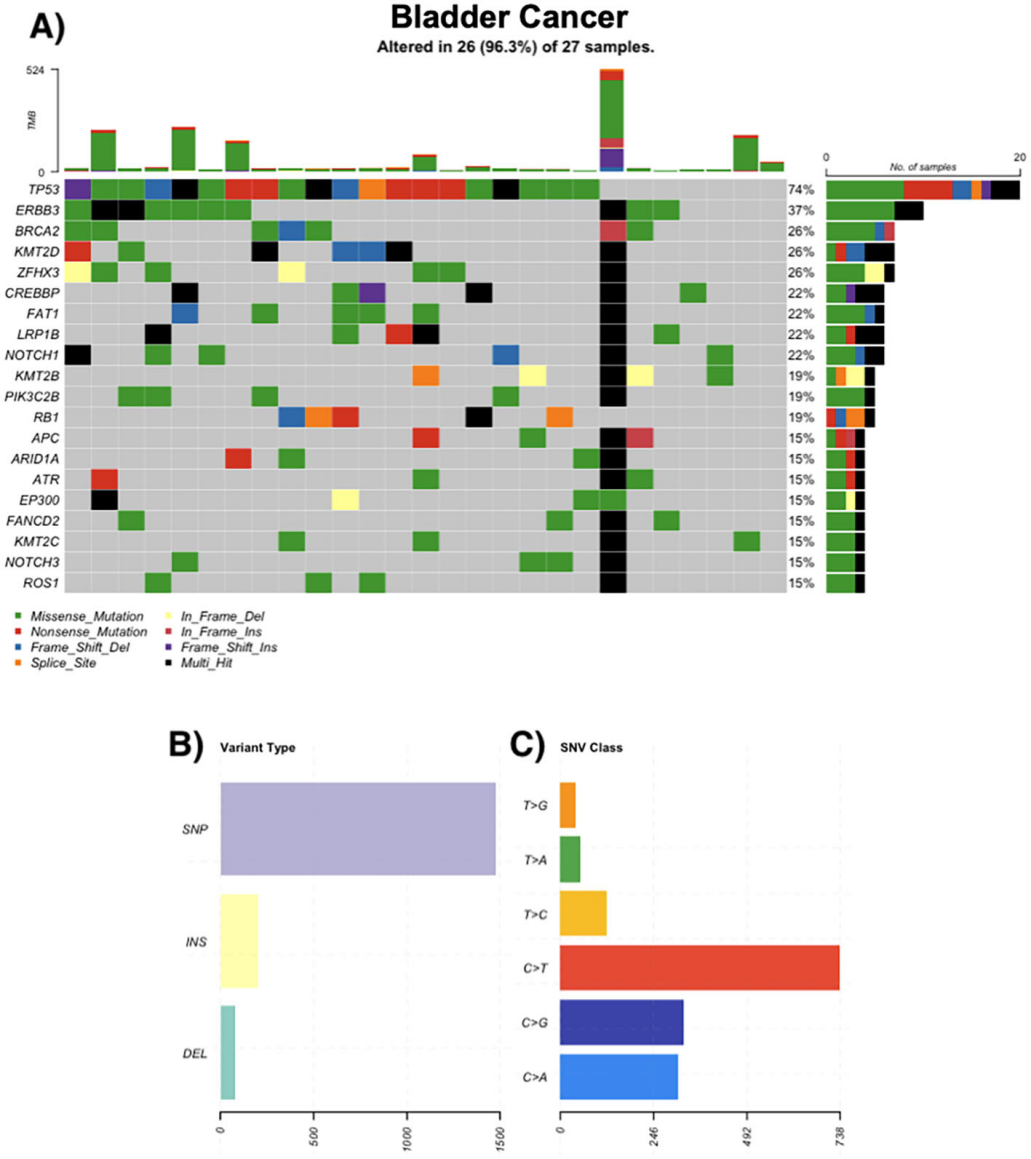
Genomic landscape of bladder cancer. **(A)** Oncoprint of recurrently altered genes in bladder tumors (n = 27); 26/27 (96.3%) samples harbor ≥1 alteration. Most frequent genes: TP53 74%, ERBB3 37%, KMT2D 26%, ZFHX3 26%, BRCA2 26%, CREBBP 22%, FAT1 22%, LRP1B 22%, NOTCH1 22%, PIK3C2B 19% (tiles by mutation class; black = multi-hit; gray = no alteration). Stacked bars above columns show TMB per sample; right-hand bars summarize the number and percentage of altered cases per gene. **(B)** Distribution of variant types across the cohort (SNPs, insertions, deletions). **(C)** Single-nucleotide variant (SNV) spectrum by substitution class (C>A, C>G, C>T, T>A, T>C, T>G); C>T transitions predominate.

Of 30 bladder tumors, 86.6% harbored mutations. Urothelial carcinoma was the most common histological subtype. Frequently altered genes included TP53 (74%), ERBB3 (37%), BRCA2 (26%), and KMT2D (26%). Mutations in chromatin regulators and DDR pathways were common. Hypermutated samples exhibited up to 524 somatic variants (**Figure 4**).

### Upper tract urothelial carcinoma

**Figure 5:**
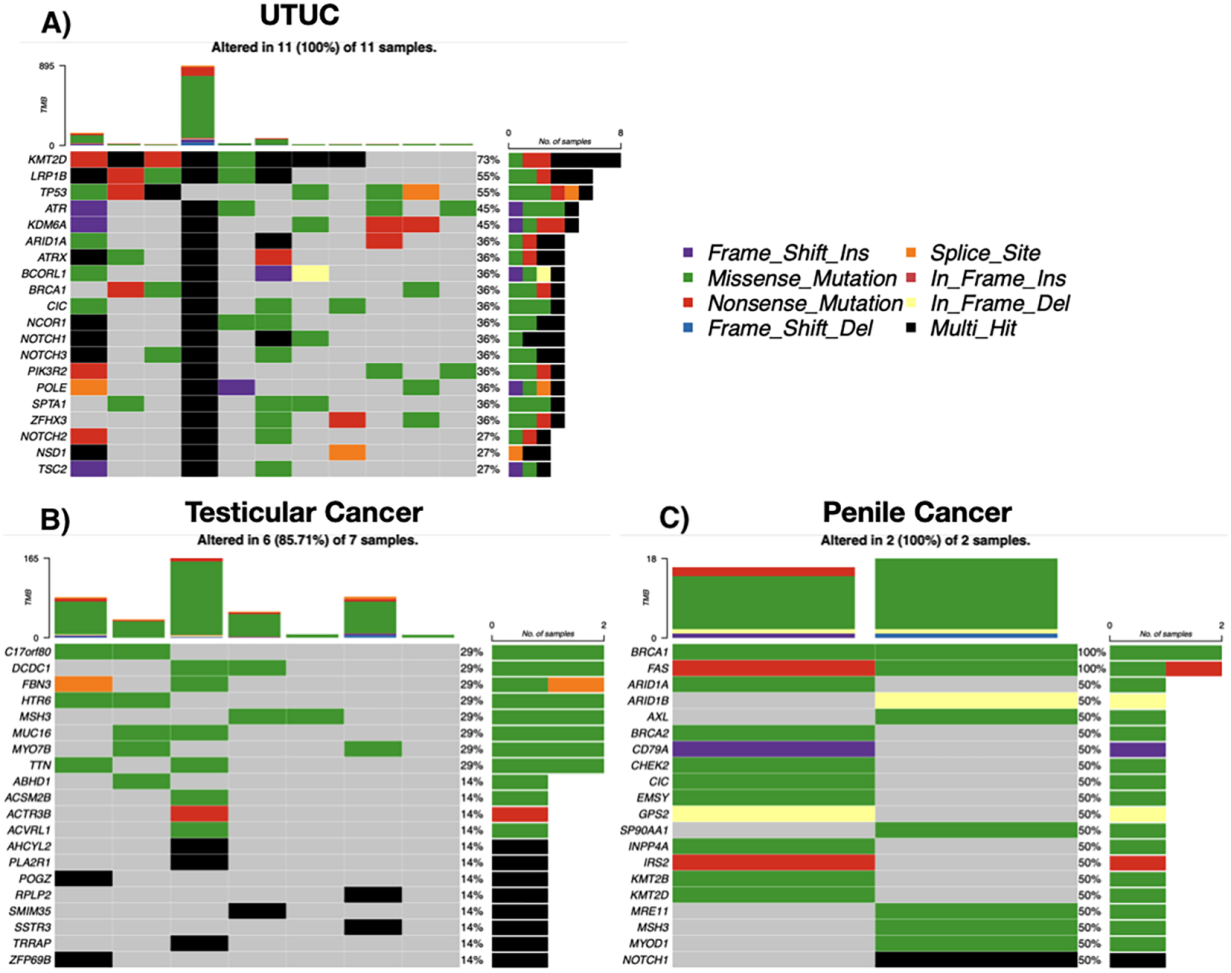
Somatic mutation landscape by tumor entity. **(A)** Upper tract urothelial carcinoma (UTUC; n = 11). **(B)** Testicular tumors (n = 7). **(C)** Penile squamous cell carcinoma (n = 2). Each oncoprint shows selected recurrently altered genes (rows) across individual tumors (columns). Colored tiles indicate mutation class (missense, nonsense, frameshift insertion/deletion, splice-site, in-frame insertion/deletion; legend), black denotes multi-hit events, and gray indicates no alteration. The stacked bar above each column depicts tumor mutational burden (TMB) for that sample, and the right-hand bars summarize the number and percentage of altered cases per gene. Percentages reflect samples with ≥1 alteration in the respective gene.

Among 12 UTUC cases, 11 (91.6%) showed somatic mutations. Recurrent alterations were seen in KMT2D (73%), LRP1B (55%), and TP53 (55%). Multiple cases exhibited high TMB, including one exceeding 800 mutations/sample. C>T transitions and chromatin modifier mutations were predominant (**Figure 5A**).

### Testicular and penile tumors

Six of seven testicular tumor cases had somatic mutations, with diverse histologies. Recurrently mutated genes included TTN, MUC16, MSH3, and ZFHX3. One case showed hypermutation (>160 mutations/sample), suggesting possible mismatch repair (MMR) deficiency. Both penile tumors (squamous cell carcinoma) exhibited mutations in BRCA1 and FAS, as well as ARID1A, CHEK2, BRCA2, and were Human Papillomavirus (HPV) positive (**Figures 5B, C**).

### MTB recommendations and clinical implementation

Of the 118 patients discussed in the MTB, 91 patients (77.1%) received at least one therapeutic recommendation based on the molecular findings. No recommendation was issued for 10 patients (8.4%), as they died prior to MTB completion. An additional 18 patients (15.2%) harbored no actionable alterations and thus received no molecularly guided treatment proposal.

Among the 91 patients who received therapeutic recommendations through the MTB, a broad spectrum of molecularly guided treatment strategies was proposed. These included targeted therapies, immune checkpoint inhibitors, antibody-drug conjugates (ADCs), and selected chemotherapy regimens, tailored to the patient’s molecular profile, tumor type, and clinical context. The most frequently recommended targeted agents were PARP inhibitors (n = 29), predominantly in tumors harboring homologous recombination repair (HRR) gene alterations such as BRCA1, BRCA2, or ATM, as well as checkpoint inhibitors (n = 28), often in cases with high TMB or microsatellite instability (MSI). FGFR inhibitors (n = 13) were suggested in patients with FGFR2 or FGFR3 mutations, particularly in urothelial and upper tract tumors, while mTOR inhibitors (n = 15) and PI3K/AKT inhibitors (n = 11) were considered for tumors with aberrations in the PI3K/AKT/mTOR pathway, including mutations in MTOR, TSC1/2, or PIK3CA. Tyrosine kinase inhibitors (n = 17) were commonly recommended in renal cell carcinoma and other entities with actionable kinase alterations or angiogenic signatures.

ADCs represented a substantial component of the recommendations, reflecting their increasing clinical relevance in urologic cancers. Sacituzumab govitecan was proposed in 38 cases, primarily for metastatic urothelial carcinoma with high TROP2 expression. Trastuzumab deruxtecan was recommended in 20 cases with HER2-positive or HER2-low expression profiles, and enfortumab vedotin in 10 patients with advanced bladder cancer expressing NECTIN-4. Additional, less frequent ADC-based recommendations included mirvetuximab soravtansine (n = 3) and disitamab vedotin (n = 1), the latter for HER2-expressing tumors. Beyond these, a diverse set of targeted therapies was advised in individual cases, including inhibitors of IDH1, KRAS, ALK, CDK4/6, WEE1, MEK, EZH2, and aurora kinases, as well as a bispecific monoclonal antibody. In a small number of patients, taxane-based chemotherapy (n = 3) or next-generation hormonal agents (n = 5) were recommended based on resistance profiles or molecular features such as AR alterations. HIF-2α inhibitors were also discussed in five RCC cases with VHL loss.

Despite the frequency and breadth of recommendations, only 17 patients (14.4%) were confirmed to have initiated therapy based on the MTB guidance, underscoring the gap between molecular findings and real-world implementation. Barriers included rapid clinical deterioration, regulatory and reimbursement hurdles, and limited access to off-label agents or clinical trials.

### Somatic interaction patterns

A correlation analysis of somatic mutations revealed multiple statistically significant co-occurrence patterns across the cohort. The strongest associations were observed between chromatin modifiers and other genomic regulators, including frequent co-mutations of KMT2C with SPTA1, SETD2, and ANKRD11. APC co-occurred with both KMT2C and BRCA2, while BRCA2 also showed enrichment alongside ANKRD11. Additional co-mutational clusters involved LRP1B with KMT2C, EP300, and ROS1, as well as RANBP2 with ROS1. FAT1 mutations were linked to both CREBBP and SPTA1, and several NOTCH pathway genes (NOTCH1, NOTCH2) displayed associations with SPTA1 and ROS1. These patterns suggest convergence within DNA damage response, chromatin remodeling, and cell signaling pathways. No statistically significant mutually exclusive mutation pairs were detected (**Figure 1**).

## Discussion

The molecular profiling of urologic malignancies included in this real-world precision oncology cohort underscores the biological diversity and clinical implications of actionable genomic alterations across different tumor entities. While urologic cancers such as prostate, bladder, renal, and rarer genitourinary tumors share certain pathways of tumorigenesis, our findings highlight unique genomic signatures with potential therapeutic relevance.

### Prostate cancer

Among patients with metastatic castration-resistant prostate cancer (mCRPC), the mutational burden and spectrum were heterogeneous. TP53 emerged as the most frequently altered gene, consistent with its known role in advanced prostate cancer and its association with poor prognosis (8). Co-occurring mutations in RB1, as identified in a subset of cases, are characteristic of neuroendocrine differentiation and resistance to androgen receptor (AR)-targeted therapies (9). The combined loss of TP53 and RB1 is a hallmark of aggressive variant prostate cancer (AVPC), necessitating platinum-based chemotherapy rather than AR-directed agents (10).

A significant proportion of mCRPC cases in our cohort harbored HRR deficiencies, particularly involving BRCA2, BRCA1, and ATM. This finding supports the routine genomic assessment of HRR genes in advanced prostate cancer, as such alterations have become important predictive biomarkers for PARP inhibitor therapy.

The clinical relevance of BRCA alterations is underscored by the PROfound trial, which demonstrated improved radiographic progression-free survival (rPFS) and overall survival in patients with BRCA1/2 or ATM mutations treated with olaparib monotherapy after progression on AR-targeted agents (11).

Beyond monotherapy, recent trials have investigated the use of PARP inhibitors in combination with androgen receptor pathway inhibitors. The TALAPRO-2 trial evaluated talazoparib plus enzalutamide as first-line therapy for mCRPC and reported a significant rPFS benefit, particularly in the HRR-deficient subgroup, but also suggested efficacy in HRR-unselected patients (12). Similarly, the MAGNITUDE trial showed improved rPFS in patients with HRR gene alterations treated with niraparib and abiraterone, supporting a combination approach in molecularly stratified populations (13).

These findings reflect a broader therapeutic paradigm in which HRR deficiency, especially BRCA2 loss, is not only an indication for PARP inhibitor monotherapy but also a rationale for upfront combination strategies in earlier lines of treatment. Mechanistically, AR signaling has been shown to regulate multiple DNA repair genes, including key components of the homologous recombination pathway (e.g., BRCA1, RAD51). Androgen deprivation reduces expression of these genes and impairs HRR proficiency, rendering tumors more susceptible to PARP inhibition—even in the absence of canonical HRR mutations (14).

As such, our observation of HRR alterations in approximately one-quarter of the prostate cancer cohort may have important implications for both current treatment eligibility and future trial enrollment, highlighting the clinical utility of comprehensive molecular profiling in this setting.

Furthermore, PTEN loss, identified in multiple cases, suggests activation of the PI3K/AKT pathway, which is targetable with AKT inhibitors such as ipatasertib. Combined blockade with abiraterone and ipatasertib has demonstrated superior antitumor activity compared to abiraterone alone, particularly in patients with PTEN-deficient tumors (15).

Although immunotherapy remains largely ineffective in prostate cancer due to its immunologically “cold” microenvironment, high TMB or mismatch repair deficiency (dMMR) may predict benefit from immune checkpoint blockade (16). The presence of outlier cases with TMB exceeding 100 mutations per megabase suggests a potential role for PD-1 inhibition in this rare subset.

### Bladder cancer and upper tract urothelial carcinoma

MIBC exhibited a high prevalence of TP53 mutations, confirming its role as a central driver of genomic instability in high-grade urothelial tumors (17). Frequent alterations in chromatin remodeling genes, including KMT2D and CREBBP, further support the involvement of epigenetic deregulation in MIBC pathogenesis. Mutations in ERBB3 and components of the PI3K pathway were also notable, suggesting therapeutic opportunities through HER and PI3K inhibition (18, 19).

In contrast, UTUC demonstrated a distinct molecular profile. KMT2D and LRP1B mutations were predominant, and a subset of tumors exhibited POLE mutations and ultramutated phenotypes, which are associated with exceptional responses to immune checkpoint inhibitors (ICIs) (20, 21). While UTUC is generally considered less immunogenic than bladder cancer, cases with high TMB or POLE mutations may benefit from ICIs. Interestingly, FGFR3 mutations—frequently observed in low-grade, papillary bladder tumors—were absent in this high-grade cohort, reflecting the inverse relationship between FGFR3 and TP53 mutation patterns (22).

These molecular differences between bladder cancer and UTUC highlight the need for tailored therapeutic strategies. Erdafitinib, an FGFR inhibitor, is approved for FGFR3-altered urothelial carcinoma, and emerging agents targeting epigenetic alterations such as ARID1A or KMT2D mutations may expand future treatment options (23–25).

### Renal cell carcinoma

The molecular landscape of RCC in this cohort was dominated by alterations in VHL, PBRM1, and SETD2—genes frequently disrupted in clear cell RCC due to chromosome 3p loss (26). BAP1 mutations define a distinct aggressive subtype characterized by poor prognosis and immune infiltration (27).

The identification of VHL mutations in approximately one-third of RCC patients reaffirms the therapeutic relevance of targeting the hypoxia-inducible factor (HIF) axis. Belzutifan, a HIF-2α inhibitor, has shown efficacy in patients with VHL disease–associated tumors and is being explored in sporadic RCC (28).

In our RCC cohort, frequent alterations in VHL, PBRM1, and SETD2 were observed. These genes are functionally linked to hypoxia signaling and chromatin remodeling, pathways implicated in both VEGF-driven angiogenesis and immune evasion. In particular, VHL loss stabilizes HIF transcription factors, leading to increased VEGF expression, which provides a mechanistic rationale for VEGF-targeted therapy (26). Simultaneously, PBRM1 loss has been associated with enhanced response to immune checkpoint blockade, potentially due to modulation of the tumor immune microenvironment. These observations support the current standard-of-care approach of combining VEGF inhibitors with immunotherapy in clear cell RCC (29).

### Penile and testicular tumors

Despite the rarity of penile squamous cell carcinoma, BRCA1 and BRCA2 mutations were identified in both cases analyzed, suggesting homologous recombination deficiency and potential sensitivity to PARP inhibitors. NOTCH1 mutations, frequently reported in HPV-negative penile tumors, were also observed (30). These alterations may serve as therapeutic targets or prognostic markers in future studies (31). Notably, both cases in our study were HPV positive.

Testicular germ cell tumors (GCTs) exhibited a markedly low mutational burden, consistent with their known genomic stability and high chemosensitivity. No classical driver mutations (e.g., KIT, KRAS) were detected, although isolated mutations in MSH3 suggest possible mismatch repair defects in rare cases. Hypermutated GCTs, while exceptional, could theoretically benefit from immunotherapy due to their increased neoantigen load; however, clinical trials have not demonstrated significant benefit of PD-1 blockade in this context (32). The role of molecular profiling in GCTs remains limited given their high cure rates with platinum-based chemotherapy.

### Somatic interaction patterns

The co-occurrence analysis of somatic alterations revealed several statistically significant mutation pairs across urologic tumor entities, suggesting biological convergence in tumor evolution. Among upper tract and bladder urothelial carcinomas, co-mutations between chromatin-modifying genes and other genes potentially indicative of structural genome instability were particularly enriched. One of the most frequent interactions was observed between KMT2C and SPTA1. While SPTA1 encodes a cytoskeletal protein, recent evidence suggests that its mutations may influence abnormal cell proliferation and apoptosis, as shown in glioblastoma studies, indicating a possible, yet incompletely understood, role in tumorigenesis (33). Additionally, KMT2C was significantly co-mutated with SETD2, ANKRD11, and APC, indicating potential convergence of mutations affecting chromatin regulation and signaling pathways. While the functional interaction of these genes in urothelial cancer remains to be clarified, their combined alteration may reflect complex epigenetic dysregulation and structural genome instability in high-grade tumors.

The tumor suppressor LRP1B, known to correlate with high tumor mutational burden and increased neoantigen load, was frequently co-mutated with KMT2C and EP300, particularly in UTUC, where both LRP1B and chromatin remodeling genes are recurrently altered (34, 35). These co-mutations may define a genomically unstable, immunoresponsive subset of UTUC.

In prostate cancer, the combination of BRCA2 and APC mutations was one of the most notable associations. BRCA2 deficiency is a well-established predictor for response to PARP inhibition, while concurrent APC mutation—leading to Wnt pathway activation—has been linked to resistance against androgen deprivation and to neuroendocrine differentiation in advanced prostate cancer (11, 36, 37). The presence of BRCA2–APC co-mutations may therefore identify a particularly aggressive mCRPC subtype that warrants combined therapeutic targeting of both pathways.

### Implementation and strategic outlook

Although molecular profiling enabled individualized therapeutic recommendations in the majority of patients, real-world implementation proved to be a substantial challenge. A large proportion of cases were presented to the MT) at an advanced disease stage, often after multiple lines of prior therapy. As a result, clinical deterioration, loss of fitness, or death before MTB completion frequently precluded the initiation of targeted treatments—even when actionable alterations were identified. When treatments were implemented, PARP inhibitors were among the most frequently used agents. Their established efficacy in prostate cancer (11–13) and regulatory approval in HRR-deficient tumors facilitated access and clinical decision-making. In contrast, other recommended therapies—such as ADCs like sacituzumab govitecan—often faced barriers related to off-label use, trial availability, or reimbursement, despite promising molecular matches.

These findings emphasize the need to initiate comprehensive molecular diagnostics earlier in the disease course, particularly upon detection of metastases or biochemical recurrence. Early testing not only increases the likelihood of clinical benefit but also improves access to ongoing clinical trials, many of which require biomarker-based eligibility. Moreover, broader awareness and institutional integration of MTBs within oncology workflows may help standardize precision oncology practices and shorten the time from sequencing to decision-making. Finally, the limited availability of approved or guideline-anchored targeted therapies in urologic oncology highlights an urgent need for drug development programs that align molecular targets with therapeutic pipelines. Ideally, such efforts should be embedded in adaptive trial designs (e.g., basket or umbrella trials) to expedite translation into clinical practice and support future evidence-based incorporation into treatment guidelines.

### Limitations

This study has several limitations that should be acknowledged when interpreting the findings.

First, the sample sizes for some tumor entities—particularly rare cancers such as penile or testicular tumors—were very small (e.g., n = 2 for penile cancer). As a result, statistical power to detect meaningful associations or derive entity-specific conclusions is limited for these subgroups. While these data contribute valuable insights into underrepresented malignancies, they should be interpreted as hypothesis-generating rather than confirmatory.

Second, the statistical analysis was deliberately limited to descriptive summaries, reflecting the heterogeneous nature and relatively modest size of the overall cohort. The primary aim of this study was to provide a real-world molecular overview of urologic tumors presented to a multidisciplinary MTB, rather than to establish predictive associations. While exploratory correlation or group comparisons may offer additional depth, we refrained from performing such analyses to avoid overinterpretation in the absence of statistical robustness. We acknowledge this as a limitation and highlight the need for larger, multi-institutional cohorts to validate and extend the patterns observed here.

Moreover, while we were able to document whether patients received therapeutic recommendations through the molecular tumor board and whether treatment was initiated accordingly, we were not able to systematically assess clinical outcomes, such as progression-free or overall survival, for the majority of cases. The lack of structured longitudinal follow-up data limits our ability to evaluate the true clinical impact of MTB-guided recommendations. We acknowledge this as an important limitation and emphasize the need for prospective outcome-based studies to determine the efficacy, durability, and real-world benefit of precision oncology strategies in urologic malignancies.

Finally, the inclusion of both panel-based and whole-exome sequencing data introduces variability in target regions and mutation detection sensitivity. Although quality control and harmonization steps were applied, differences in sequencing coverage and platform resolution may affect comparability, particularly with respect to TMB and low-frequency variants.

Together, these limitations underscore the exploratory nature of this study and the importance of contextualizing its findings within a broader framework of ongoing precision oncology research.

## Conclusion

This real-world molecular analysis highlights the genomic complexity and clinical heterogeneity of urologic malignancies. By integrating comprehensive sequencing data into a multidisciplinary MTB framework, actionable insights were generated across both common and rare tumor types. The observed co-mutational patterns suggest that beyond single-gene alterations, combinatorial genomic signatures may better inform targeted therapy and trial enrollment. These findings reinforce the value of precision oncology approaches in guiding personalized treatment for patients with advanced or refractory urologic cancers.

## Data Availability

The data analyzed in this study is subject to the following licenses/restrictions: Patient consent and the ethics approval cover only the predefined research purpose within our institution; they do not permit external or public sharing. Due to small cohort size and rare entities, the re-identification risk remain non-negligible even after pseudonymization. Requests to access these datasets should be directed to maximilian.glienke@uniklinik-freiburg.de.
